# 非小细胞肺癌寡转移的研究进展及挑战

**DOI:** 10.3779/j.issn.1009-3419.2025.102.22

**Published:** 2025-06-30

**Authors:** Songzhen LI, Tianhang SHAO, Shuyang YAO

**Affiliations:** ^1^100053 北京，首都医科大学第一临床医学院（李松珍，邵天航）; ^1^The First Clinical Medical College, Capital Medical University; ^2^首都医科大学宣武医院胸外科（姚舒洋）; ^2^Department of Thoracic Surgery, Xuanwu Hospital, Capital Medical University, Beijing 100053, China

**Keywords:** 肺肿瘤, 寡转移, 综合治疗, Lung neoplasms, Oligometastasis, Comprehensive treatment

## Abstract

寡转移是早期转移和广泛转移之间的过渡状态，肿瘤负荷有限，具备独特的肿瘤生物学行为。由于转移灶的数量和受累器官的数量相对有限，经过积极的系统治疗和局部巩固治疗，有潜在治愈的机会。随着分子靶向药物治疗和免疫治疗的飞速发展，非小细胞肺癌（non-small cell lung cancer, NSCLC）寡转移的综合治疗越来越受到重视。本文对NSCLC寡转移的定义、治疗进展以及存在的诸多挑战进行综述，以期为临床诊疗提供思路。

## Abstract (en)

Oligometastasis represents a transitional state between early localized disease and widespread metastasis, characterized by limited tumor burden and distinct tumor biological behavior. Due to the relatively restricted number of metastatic lesions and involved organs, aggressive systemic therapy combined with local consolidative therapy offers potential for cure. With rapid advancements in molecular targeted therapies and immunotherapy, comprehensive management of oligometastatic non-small cell lung cancer (NSCLC) has gained increasing attention. This review summarizes the definition of NSCLC oligometastasis, recent therapeutic progress, and existing challenges, aiming to provide insights for clinical diagnosis and treatment strategies.

非小细胞肺癌（non-small cell lung cancer, NSCLC）是一种主要的肺癌亚型，约占所有肺癌的85%，严重威胁人类健康^[1]^。超过一半的患者在初次诊断时已经出现远处转移，这表明疾病处于IV期，在绝大多数情况下被认为是无法治愈的。1995年Hellman和Weichselbaum共同提出了“寡转移”的概念，用以描述数量及分布有限的远处转移性疾病状态，它是肿瘤自然发展过程中的中间阶段，介于局限性原发肿瘤与广泛转移性疾病之间（肿瘤负荷有限，且具备独特的肿瘤生物学行为）^[2]^。

长期以来，普遍认为无论对寡转移还是广泛转移病灶进行的局部治疗都仅是姑息作用。在认识到寡转移的概念后，癌症患者的目标和治疗策略发生了巨大的变化，即从改善症状的姑息性治疗转变为以治愈为目的的根治性治疗^[3]^。NSCLC寡转移的证据已逐步积累：早期证据均来自回顾性研究或小型单臂II期临床试验和系统综述，而近期随机对照的II或III期试验进一步证实了对所有病灶采取局部积极治疗的患者，其无进展生存期（progression-free survival, PFS）和总生存期（overall survival, OS）均获得显著改善^[4]^。

在这篇综述中，我们将重点探讨NSCLC寡转移患者最具争议性的几个方面，包括（但不限于）：该状态的定义、系统性治疗与局部巩固治疗（local consolidative therapy, LCT）的联合方案、分子标志物的探索等关键问题。

## 1 寡转移的基本概念

目前尚难以对NSCLC寡转移进行明确定义，不同学者界定转移病灶数量和受累器官数目的标准存在显著不同。对于是否纳入特定部位（如颅内病灶或纵隔淋巴结转移）的转移患者，各研究也存在分歧^[5]^。欧洲放射治疗与肿瘤学学会（European Society of Therapeutic Radiology and Oncology, ESTRO）的OligoCare项目致力于为所有原发肿瘤类型定义具有生物学差异的寡转移疾病状态。该项目根据以下因素对各类寡转移疾病进行分类：寡转移诊断与原发肿瘤诊断的时间关系、是否正在接受全身治疗、既往是否接受过寡转移或广泛转移的治疗，以及疾病对既往或当前治疗的反应。

现行肿瘤原发灶-淋巴结-转移（tumor-node-metastasis, TNM）分期体系将M1a期定义为存在胸膜转移或对侧肺转移（无胸外转移），M1b期为单发胸外转移，而M1c期则包括单器官或多器官的多发胸外转移^[6]^。然而现有文献对寡转移的定义与该分期系统并不完全吻合，一项系统综述^[5]^显示，各研究中纳入寡转移病例的转移灶数量上限从1-8个不等，受累器官最多为3个。欧洲多学科专家组达成的共识，将寡转移性疾病定义为不超过3个器官的5处转移灶，且除外脑膜、心包、胸膜或肠系膜等部位的弥漫性浆膜转移，或存在骨髓浸润。但该定义尚未获得普遍认可，仍需更多研究来标准化寡转移概念。寡转移NSCLC的发病率估计在27%至55%之间^[7]^。最常见的寡转移部位为脑部（36%），其次是对侧肺（34%）、肾上腺（13%）、骨骼（9%）和肝脏（2%）^[8]^。寡转移可能比广泛转移具有更惰性的生物学特性，或至少是可通过全身治疗根除的微小病灶。这种有限的转移表型可能被称为LCT的局部积极治疗所治愈。

随着LCT技术的持续革新与进步[微创手术技术、加速康复外科路径以及更精确的放疗技术，如立体定向体部放疗（stereotactic body radiotherapy, SBRT）、门控放疗等]，LCT安全且耐受性良好，因此，治疗可行性的定义可能变得更为模糊。这一点至关重要，因为多项研究证实与对原发灶及所有转移灶均实施彻底性LCT治疗的患者相比，仅对部分（而非全部）转移灶进行非彻底性LCT治疗的患者生存结局更差^[9-11]^。

## 2 同时性寡转移与异时性寡转移

转移灶出现的时机可能影响寡转移NSCLC患者的预后判断^[8]^。同时性寡转移指原发肿瘤确诊时即被发现的转移灶，而异时性寡转移则是在原发肿瘤治疗后出现的转移灶^[12,13]^。目前文献对同时性寡转移与异时性寡转移之间的时间间隔尚无明确定义，且各研究间存在较大差异^[8,12,14]^。不过，根据欧洲癌症研究与治疗组织（European Organization for Research and Treatment of Cancer, EORTC）-ESTRO共识推荐对寡转移疾病的分类标准，将原发肿瘤诊断后6个月作为区分时点：超过6个月出现的转移灶归为异时性，6个月及以内出现的则视为同时性。

这两种类型可能代表了不同的生物学进程，接受LCT后的预后也存在差异。同时性寡转移可能意味着疾病已进入更晚期的恶性转移阶段，这提示可能存在隐匿的广泛微转移病灶。而异时性寡转移则更可能代表早期的转移潜能，是在原发肿瘤治疗后，由少数具有转移潜能的细胞克隆扩增形成的^[15]^。一项大型个体数据荟萃分析^[8]^发现，与同时性寡转移相比，异时性寡转移患者接受LCT后的OS获益更大。目前支持LCT用于寡转移性NSCLC的前瞻性数据主要来自同时性寡转移疾病，而专门针对异时性寡转移NSCLC的前瞻性数据则较为有限^[15]^。因现有文献对此结论并不完全一致，仍需进一步研究。

## 3 治疗策略的优化

目前的研究大多为寡转移NSCLC患者在系统性治疗至少达到疾病稳定的状态后，再对所有病灶进行LCT。而且，LCT起到了关键作用，其主要手段包括手术切除、放射治疗^[16]^以及射频消融技术^[17]^。系统性治疗包括用于NSCLC治疗的免疫检查点抑制剂（immune checkpoint inhibitors, ICIs）治疗、靶向治疗和化疗等。

SINDAS^[18]^是一项III期随机对照试验，旨在评估放疗联合酪氨酸激酶抑制剂（tyrosine kinase inhibitors, TKIs）一线治疗在初治寡转移性表皮生长因子受体（epidermal growth factor receptor, EGFR）突变NSCLC患者中的疗效。共133例入组，65例患者只接受TKIs治疗，68例患者接受TKIs联合放疗。结果显示TKIs联合放疗组的中位PFS为20.2个月，明显优于TKIs组的12.5个月（HR=0.22, 95%CI: 0.17-0.46），相较于TKIs组，TKIs联合放疗组的中位OS也有显著延长（25.5 vs 17.6 mon, HR=0.44, 95%CI: 0.28-0.68）。治疗的耐受性良好，没有出现5级不良反应（adverse event, AE），TKIs联合放疗组中，3-4级有症状的肺炎发生率为6%。

CURB试验^[19]^是一项II期随机对照临床研究，旨在评估SBRT联合标准的系统性治疗对寡进展性NSCLC或乳腺癌患者的疗效。该研究共纳入106例患者，其中乳腺癌47例，NSCLC 59例。在NSCLC患者中，31例接受SBRT治疗，28例接受标准的系统性治疗。结果显示：联合治疗组中位PFS为10.0个月（95%CI：7.2-未达到），明显优于标准治疗组的2.2个月（95%CI: 2.0-4.5）。SBRT可显著延长NSCLC患者PFS，但遗憾的是，在乳腺癌患者中未观察到相似的疗效提升。

NRG-LU002试验^[20]^是一项II/III期随机对照临床研究，旨在评估局部LCT联合维持性全身治疗对寡转移NSCLC患者的疗效。该研究专门纳入初始全身治疗（化疗或ICIs）后达到至少疾病稳定的且具有≤3个颅外转移灶的患者。共纳入215例患者，其中81例进入单纯维持治疗组，134例进入LCT联合维持治疗组。主要终点为PFS，试验设计要求仅在II期试验观察到显著获益时才进入III期阶段。研究结果显示两组间PFS无统计学显著差异（HR=0.93, 95%CI: 0.66-1.31），两组的OS同样未见差异（HR=1.05, 95%CI: 0.70-1.56）。LCT联合组≥2级AE率更高（84% vs 73%），且≥3级肺炎发生率显著增加（10% vs 1%）。该试验是迄今规模最大的寡转移NSCLC II/III期临床研究，值得注意的是，该试验中超过90%的患者接受了以ICIs为基础的系统治疗，体现了当前的临床实践，但这一特征与既往研究亦存在本质差异。亚组分析或可解释该研究结果与既往研究的差异。关于该队列未观察到LCT获益的可能机制包括：ICIs疗效的提升削弱了LCT的作用价值、前期ICIs后残留病灶的特殊生物学特性，以及研究组间潜在的人群差异。虽然目前仅公布了初步数据，但这些结果提示LCT不应作为常规治疗推荐，而应通过多学科讨论根据个体化情况做出决策。在权衡治疗方案时，需综合评估治疗风险与病灶进展可能引发的症状或AE风险。

CURE-OLIGO试验^[21]^是一项针对适合接受胸部病灶根治性放化疗（chemoradiotherapy, CRT）的IV期寡转移NSCLC患者开展的多中心II期单臂临床试验。治疗方案为采用含铂双药方案的胸部病灶CRT治疗，并在开始或完成CRT后8周内对寡转移灶进行LCT。主要研究终点为2年生存率。共入组19例患者，有效率为58%，中位PFS和中位OS分别为8.6个月（95%CI: 7.0-10.2）和42.1个月（80%CI：13.6-未达到）。2年生存率为68.4%（80%CI: 52.6%-79.9%）。没有出现新的安全性信号，耐受性良好。

## 4 分子标志物的探索

目前缺乏可靠的生物标志物预测哪些患者能真正从系统性治疗联合LCT中获益。部分患者接受LCT后仍快速进展，提示可能存在隐匿性微转移。

循环肿瘤DNA（circulating tumor DNA, ctDNA）作为一种极具前景的生物标志物，是一种筛选适合系统性治疗联合LCT患者的有效工具。一项纳入309例寡转移NSCLC患者的多中心队列研究^[22]^显示：放疗前可检测到ctDNA的患者，PFS和OS显著差于ctDNA阴性者。这一发现证实ctDNA可作为风险分层工具，识别存在微转移灶、可能更适合系统性治疗而非局部巩固放疗的患者群体。此外，ctDNA对根治性放疗后的微小残留病灶也具有预后价值。纵向监测数据^[23]^显示，治疗后ctDNA阳性可预测复发风险。因ctDNA检测技术尚存在低丰度ctDNA导致的假阴性结果、克隆性造血相关突变导致的假阳性结果、检测技术标准化与质控的困境、临床证据的异质性等问题，需要在大规模前瞻性寡转移NSCLC患者的研究中进一步探索动态监测ctDNA作为分子标志物的可行性。

## 5 挑战与机遇

系统性治疗联合LCT的综合治疗模式纳入临床实践主要面临三大挑战：患者筛选、新技术整合及毒性的控制。由于并非所有寡转移NSCLC患者都能从中获益，精准选择治疗人群至关重要。虽然数项研究发现系统性治疗联合LCT的综合治疗有助于改善患者的PFS，但是存在更广泛微转移的患者可能无法从这种治疗策略中获益。2024年美国临床肿瘤学会（American Society of Clinical Oncology, ASCO）大会上公布的NRG LU002研究^[20]^没有见到联合治疗对患者的帮助，因此该研究提前终止且未进入III期研究阶段。不同患者群体从LCT中获益的程度可能存在显著差异。例如，对系统治疗反应良好的患者可能从LCT获益有限，因为持续的系统治疗本身已能有效控制疾病；而系统治疗效果不佳的患者，则可能通过LCT获得更大收益。因此，我们需要更多针对新型治疗方案下寡转移NSCLC患者的临床试验来评估SBRT的价值，鉴于早期试验数据可能已不适用于当前治疗格局。

精确分期和筛选适合巩固性放疗的患者需依赖正电子发射计算机断层显像（positron emission tomography/computed tomography, PET/CT）等现代影像技术，PET/CT能有效鉴别寡转移与广泛转移。此外，控制放疗毒性对保障患者生活质量至关重要。维持化疗联合巩固性SBRT未显著增加严重毒性，但放射性肺炎/食管炎等副作用仍是临床挑战和限制性因素。

探索质子治疗和超高速高剂量率（fast light application in stochastic high-dose-rate, FLASH）放疗等新型放射治疗技术，为优化治疗方案提供了新机遇。其中，FLASH放疗能实现超高剂量率照射，在最大化肿瘤控制的同时展现出降低毒副作用的潜力^[24]^。此外，Jazieh团队^[25]^的研究发现基于治疗前CT扫描的影像组学特征可预测放化疗联合度伐利尤单抗治疗患者的PFS和OS，影像组学高风险评分与不良预后显著相关。但仍需要更多更扎实的研究来进一步证实新的LCT技术在寡转移NSCLC治疗中所起的作用。

## 6 总结

寡转移是早期转移和广泛转移之间的过渡状态。寡转移NSCLC的定义虽尚未统一，但越来越多的临床研究^[3,26]^表明对这部分患者进行有效的系统性治疗后并对所有残余病灶彻底实施LCT，可有效控制肿瘤负荷，明显提高生存时间。微创手术以及SBRT等现代放疗技术的应用，为精准治疗策略提供了技术支持。然而，在新型系统性治疗下优化患者筛选和应对治疗相关毒性方面存在新的巨大挑战。影像学技术的改进和预测性生物标志物的发展有助于筛选可能从LCT中获益的患者。未来研究将聚焦于优化治疗方案、开发创新LCT技术，以提升治疗精度并减少AE。
